# Determinants of delayed care seeking for TB suggestive symptoms in Seru district, Oromiya region, Ethiopia: a community based unmatched case-control study

**DOI:** 10.1186/s12879-017-2407-8

**Published:** 2017-04-20

**Authors:** Robel Yirgu, Firaol Lemessa, Selamawit Hirpa, Abraham Alemayehu, Eveline Klinkenberg

**Affiliations:** 1 Department of Reproductive Health and Health Service Management, School of Public Health, College of Health Sciences, Addis Ababa University, Addis Ababa, Ethiopia; 2 Department of Public Health, College of Health Sciences, Arsi University, Rustavi, Georgia; 3 Arsi Zone Health Bureau, Seru District Health Office, Seru Health Center., Seru, Ethiopia; 4 KNCV Tuberculosis Foundation, The Hague, Netherlands; 5 Department of Global Health, Academic Medical Centre, Institute for Global Health and Development, University of Amsterdam, Amsterdam, Netherlands

**Keywords:** TB, Patient delay, Care seeking

## Abstract

**Background:**

Early tuberculosis (TB) case finding and adequate chemotherapy are essential for interrupting disease transmission and preventing complications due to delayed care seeking. This study was undertaken in order to provide insights into the magnitude and determinants of patient delay.

**Methods:**

The study was conducted in rural Seru district, employing a population based unmatched case-control study design. The WHO standardized TB screening tool was used to identify presumptive TB cases among the district population ages > 15 years. Presumptive TB cases who sought care in a health facility more than 14 days after the onset of symptoms were considered cases while those who sought care within the first 14 days were classified as controls. A structured interview questionnaire was used to capture socio demographic characteristics and health care service utilization related data from the study participants. A multiple binary logistic regression model was used to identify any factor associated with patient care seeking delay.

**Result:**

A total of 9,782 individuals were screened, of which 980 (10%, 95% CI; 9.4-10.5%) presumptive TB cases were identified. From these cases 358 (76%, 95% CI; 75.6%-76.4%) sought care within the first 14 days of the onset of symptoms with a median patient delay of 15 days, IQR (5-30 days). The most common TB suggestive symptom mentioned by the participants was night sweat 754 (76.4%) while the least common was a history of contact with a confirmed TB case in the past one year 207 (21.1%). Individuals in the 45-54 age range had lower odds of delay (AOR 0.31, 95%CI 0.15, 0.61) as compared to those 15-24 years old. First TB treatment episode (AOR16.2, 95% CI 9.94, 26.26) and limited access to either traditional or modern modes of transportation (AOR 2.62, 95% CI 1.25, 5.49) were independently associated with patient care delay.

**Conclusion:**

Increasing community awareness about the risks of delayed care seeking and the importance of accessing health services close to the community can help decrease patient care delay.

## Background

Early identification and adequate treatment of infectious tuberculosis (TB) patients is mandatory in reducing transmission and ultimately elimination of the disease [1]. Early case finding and adequate chemotherapy with a standardized combination of drug regimens remains top priority for curing patients, interrupting transmission to other contacts and preventing complications associated with advanced disease [2]. In most developing countries, including Ethiopia, passive case finding is the main approach for identifying TB cases [3]. In passive case finding, patients who are aware of their symptoms are expected to approach the health system for diagnosis and treatment. This approach is less expensive and conforms to the customary patient-health system relationship, whereby patients take the initiative to visit health care facilities. The effectiveness of passive case finding is determined by both health system and patient related factors. Thus, TB control programs in high TB burden countries like Ethiopia can benefit from a clear understanding of the factors which determine health care seeking for TB diagnosis and treatment [3, 4]. Generally, in low income and middle income countries (LMIC) the median patient delay is 31.7 days, but country level estimates indicate even more protracted delays [3, 5, 6]. Factors such as; age, sex, marital status, income and availability of health insurance, reported stigma and occupation can affect patient delay [6, 7]. In Ethiopia, according to facility based studies conducted in the Addis Ababa and Tigray regions, total delay (patient and health system delay) ranges from 64 to 99 days, while the median patient delay is 60 days [6, 8]. Even though patient delay contributes to more than half of the total delay, most in country studies are facility based, collecting data from patients who seek care from health facilities. Recognizing the inherent difference between patients at health care facilities and people with TB suggestive symptoms in the community, this community based study took the added step of identifying determinants of patient delay from the perspective of presumptive TB cases in their home setting.

## Methods

### Study area

The study was conducted in Seru district, Arsi zone, Oromiya regional state, Ethiopia. Arsi zone is located 175 kilometers south east of the capital city, Addis Ababa and is comprised of 25 districts; 1 urban and 24 rural. The zone is among the most populous zones in Oromiya Region with 2,637,657 people in an area of 21,008 km^2^ resulting in a population density of 148 people per km^2^ [9, 10]. Seru district was selected for this study because it is one of the remotest districts in the zone and had the lowest TB case finding performance according to the district’s annual performance plan for 2013 [11]. In the district, there are 14 kebeles (Ethiopia’s smallest administrative unit), with a total population of 47,979. At the time of the study, there were two government run health centers in the district, both providing directly observed therapy short-course (DOTS) services. Every kebele has one health post, serving 2500-5000 people each [10]. Health posts are primary health care facilities, staffed with 1 to 2 health extension workers (HEWs). In order to reduce TB transmission HEWs are trained to give health education on TB and identify and refer presumptive TB cases to nearby health centers [12].

### Study design and sample selection procedure

This community based unmatched case control study was conducted from June to July 2014. First, all individuals aged >15 years living in the study sites were screened for TB suggestive symptoms. A presumptive TB case was defined as an individual who responded yes to at least one of the symptoms listed in the WHO standard TB screening tool [13]. The tool contains five TB suggestive symptoms: cough > 2weeks, fever > 2weeks, night sweats > 2weeks, sudden weight loss in last 4 weeks and living with or having a close contact with a known TB patient in the past 1 year.

**Fig. 1.**
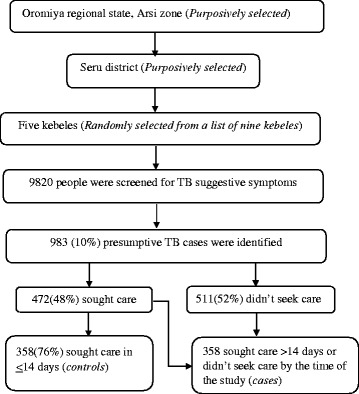
Sample selection procedure

Guidelines recommend that care should be sought within the first 2 to 3 weeks from the onset of symptoms [14]. Based on this standard, we defined ‘delay’ as seeking care more than 2 weeks after the onset of symptoms. In our study, ‘cases’ were defined as presumptive TB cases who delayed seeking care from public or private health care facilities, including health posts, within the first 14 days. Controls were defined as presumptive TB cases who sought medical care within the first 14 days since the onset of the patient’s first symptom (Fig. 1).

### Sample size calculation

A two population proportion formula with 95% confidence level and 80% power was employed to determine an optimal sample size. Inadequate knowledge about TB was included as a key variable with an anticipated prevalence of 11.9% among cases and 6.3% among controls [15]. Taking a 1:1 case-control ratio a total sample size of 716 (358-cases and 358-controls) was calculated. Assuming an 8.6% prevalence of presumptive TB cases in the community [16], the total number of participants needed for screening in order to obtain the required number of cases and controls was estimated at 9820.

Seru district has a total of 14 kebeles but due to geographic inaccessibility five kebeles were excluded from the study. From the remaining 9 kebeles five were randomly selected to obtain the sufficient number of participants for screening. The screening was conducted among all residents of the five selected kebeles. Based on the data from the screening a sampling frame containing a list of presumptive TB cases was prepared and study participants were randomly selected from the frame.

### Data collectors and data collection procedure

Data was collected by 12 high school level education complete data collectors under a close supervision from a supervisor and the study team. Each of the data collectors received a 3-day training on the data collection tools and data collection approach. Both the screening tool and interview questionnaires were translated to the local Afan Oromo language. The interview questionnaire was pretested in the neighboring Robe district before the actual data collection.

Screening data was collected from all eligible participants, in the selected kebeles, by using the screening tool. Data collectors went house to house to collect data; when participants were not found during the first visit, two more subsequent visits were made before excluding them from the survey. The interview with case and control group participants was conducted after selecting patients based on the screening result. Socio demographic and health care service utilization related variables such as; age, sex, marital status, level of education, occupation, ever been tested for HIV, history of previous TB treatment, perceived distance from the nearest health care facility and mode of transportation to the facility were all included as variables on the interview questionnaire (Table 1).

**Table 1:** List of variables and operational definition Arsi zone, Ethiopia, June 2014.

Variables	Definition
	Operational definition (categories)
Education	Level of education the participants had at the time of the study (1= Can’t read and write; 2= Can read and write, but haven’t had formal schooling; 3= Primary education, from grade 1 to grade 8; 4= Secondary education, grade 9 and 10; 5= Above secondary includes college preparatory (grade 11 and 12), technical and vocational and university.)
Occupation	The main income generating activity. (1= No job, participant who is not taking part in income generating activities and not attending classes for those >18 years old; 2= House wife, only if engaged in domestic chores activities)
Owning land for agriculture	Permanent or temporary land ownership status at the time of the study.
Perceived geographic access	Participants perception of the distance between his/her residence and the nearest health facility.

### Statistical analysis

Data was entered using *EpiData version.3.1.* statistical software, and exported to Statistical Package for Social Sciences (SPSS) *Version 20* software for further analysis.

The proportion of delayed care seeking was calculated taking presumptive TB cases who sought care after 14 days from onset of the first symptom as a numerator and all presumptive TB cases as a denominator. Similarly, the prevalence of TB suggestive symptoms was calculated by dividing the number of presumptive TB cases with the total number of individuals 15 years and older who were screened in the selected kebeles. Frequency and proportion of socio demographic and health care service related variables were analyzed in a similar manner.

Simple binary logistic regression was used to measure the association between different socio demographic and health service related variables and care seeking delay.

A multiple binary logistic regression model was used to measure the independent association between care seeking delay and various predictors by controlling for the effect of potential confounding variables. Level of significance was set at a p-value of 0.05.

## Results

### Participant profile

A total of 9,782 individuals were screened for TB suggestive symptoms of which 983 (10%, 95%CI; 9.4%-10.5) were presumptive TB cases. From these cases, 472(48%, 95%CI; 44.9%-51%) sought medical care, where 358(76%, 95% CI; 75.6%-76.4%) of them sought care in the first 14 days. All the selected cases and controls agreed to participate in the study resulting in a response rate of 100%. The median patient delay was 15days, IQR (5-30days). The most frequently reported TB suggestive symptom was night sweat 754 (76.4%). Contact history with a confirmed TB case in the past one year was reported by 207 (21.1%) participants (Table 2).

**Table 2:** Socio demographic characteristics and distribution of TB suggestive symptoms among presumptive TB cases, Arsi zone, Ethiopia, June 2014 (n=983).

Variable	Presumptive TB cases No (%)
Age (yrs)	
15- 25	345 (35.1)
26-35	209 (21.3)
36-45	178 (18.1)
46-55	124 (12.6)
>56	127 (12.9)
Median age^a^	36.3+ 16.7
Sex	
Male	374(38.0)
Female	607(61.7)
TB suggestive symptoms	
Cough of more than 14 days	671(68.3)
Night sweat	754(76.4)
Fever> 2 weeks	665(67.7)
Weight loss>3kgs in 4 weeks	581(59.1)
Contact history with a confirmed TB case in the past 1 year	207(21.1)

^a^Median age + SD

### Factors associated with patient delay

In the bi-variate analysis access to any mode of transportation other than ‘on foot’, previous history of TB treatment and HIV testing were significantly associated with delayed care seeking. Participants in 45 to 54 year age range sought medical care more often in the first 14 days (AOR, 0.31 95% CI 0.15, 0.61) compared to those in the range of 15 to 24. Those who did not have access to any mechanized means of transport were 2.6 times more likely to delay beyond 14 days (AOR, 2.62 95% CI 1.25, 5.49). The odds of delay among participants without previous history of TB treatment as compared to their counterparts was (AOR, 16.16 95% CI 9.94, 26.26) (Table 3).

**Table 3:** Factors associated with patient delay Arsi zone, Ethiopia, June 2014(n=716).

Variables	Cases(n=358) No (%)	Controls(n=358) No (%)	Crude OR (95% CI)	Adjusted OR (95% CI)
Age				
15-24	103(28.9)	81(22.4)	1	1
25-34	85(23.8)	76(21.3)	0.86(0.56,1.32)	0.81(0.44,1.47)
35-44	78(21.8)	72(20.2)	0.83(0.54,1.29)	0.53(0.28,1.01)
45-54*	39(10.9)	72(20.2)	0.42(0.26,0.68)	0.31(0.15,0.61)
55-64	26(7.3)	31(8.7)	0.65(0.36,1.17)	0.88(0.39,1.99)
>65	26(7.3)	26(7.3)	0.77(0.42,1.43)	0.71(0.29,1.69)
Sex				
Male	135(37.7)	145(40.5)	1	
Female	223(62.3)	213(59.5)	1.13(0.84,1.53)	
Education				
Illiterate	189(52.8)	184(51.4)	1	
Read and write	52(14.5)	65(18.2)	0.78(0.51,1.18)	
Primary	82(22.9)	73(20.4)	1.09(0.75,1.59)	
Secondary	27(7.5)	29(8.1)	0.91(0.52,1.59)	
Above secondary	8(2.2)	7(2.0)	1.11(0.39,3.13)	
Marital status				
Never married	77(21.5)	65(18.3)	1	
Married	254(70.9)	264(74.4)	0.81(0.56,1.18)	
Widowed	27(7.5)	26(7.3)	0.88(0.47,1.65)	
Household size				
< 5	147(41.3)	166(47.3)	1	
> 5	209(58.7)	185(52.7)	1.28(0.95,1.72)	
No of rooms				
<2	281(78.5)	281(78.5)	1	
> 2	77(21.5)	77(21.5)	1.00(0.70,1.43)	
Owning radio				
Yes	119(33.3)	142(39.9)	1	
No	238(66.7)	214(60.1)	1.33(0.98,1.80)	
Land ownership				
Yes	300(83.8)	289(81.0)	1	
No	58(16.2)	68(19.0)	0.82(0.56,1.21)	
Perceived geographic access				
Near	155(43.3)	108(30.2)	1	
Far*	203(56.7)	250(69.8)	0.57(0.42,0.77)	
Mode of transportation				
Traditional or modern	17(4.8)	49(13.7)	1	1
On foot*	337(95.2)	309(86.3)	3.14(1.77,5.58)	2.62(1.25,5.49)
Previous TB treatment				
Yes	28(8.0)	197(55.3)	1	1
No*	324(92.0)	159(44.7)	14.34(9.24,22.23)	16.16(9.94,26.26)
Ever tested for HIV				
Yes	144(40.2)	174(48.6)	1	
No	214(59.8)	184(51.4)	1.41(1.05,1.89)	

Symbols indicate p-values in the bivariate analysis: * *P*<0.001

## Discussion

### Treatment seeking delay

In our study the median days of patient delay before first consultation was 15 days, IQR (5-30). Among the presumptive TB cases who sought care, 358 (76, 95 CI; 75.6-76.4%) of them sought care within 14 days after onset of the first symptom. Controlling for the effect of socio demographic and health care service related variables, age of the participant, access to any mode of transportation other than ‘on foot’ and having a previous history of TB treatment showed a significant association with patient delay. Individuals aged 45-54 years tend to seek care earlier than those in the range of 15 to 24 years. Those who did not have access to any modes of transportation other than ‘on foot’ and those without a previous history of TB treatment were more likely to seek care after being symptomatic for at least 2 weeks.

The 15 days median patient delay is fairly modest in comparison with findings from other studies in Ethiopia. According to a study done in Amhara region the median delay was 30 days and a study in Somali region reported a median delay of 34 days and in the capital Addis Ababa 60 days [5, 7, 15, 17]. The observed shorter delay in our study could be due to the fact that our study was a community based study which included discussions with health extension workers in the patients’ villages, within the definition of a ‘first consultation’. Facility based studies often consider the day the patient appeared at the institution as the day of first consultation, therefore a patient who passes through the referral system is considered delayed when reaching the ultimate health care provider.

Patients visit health care facilities when they perceive their symptoms as serious, this prolongs their stay with the symptoms before seeking care [8]. This justifies the need to strengthen community based TB control programs in order to reach patients at a house hold level and promote health care seeking. The difference in livelihood (the fact that the Somali region is a pastoralist area) and the relative limited access to public health facilities by the time the study in Somali region was conducted can explain the longer patient delay as compared to this study [15].

### Determinants of patient delay

Different studies observed that advanced age is one of the predictors of patient delay [18, 19]. However, in our study individuals in the 45-50 year old age range sought care earlier than those 15-24. In this farming community, delayed care seeking among those 15-24 years could be explained by the possibility that minors cannot decide when to seek heath care on their own. The quoted articles which observe delays among older age groups tend to use an open-ended reference age category of (>45 years). This category can possibly include very old individuals who may need physical and financial assistance from others to seek care, this could partly explain the observed difference. This argument is supported by studies where the odds of delay are significantly high among individuals of age >60 years [3, 20].

Individuals with a previous history of TB treatment were 16 times more likely to seek care earlier than those who were being treated for the first time. This can be attributed to the prior exposure to the health system during their previous visits and potentially increased knowledge about the dangers of delayed care seeking. A better knowledge, secondary to the exposure, about the availability, costs and logistics of TB treatment services at health facilities can also minimize delays [17].

Mode of transportation was also predictive of patient delay. Those who had access to modern or traditional means of transportation had a better chance of seeking care earlier than those who had to go on foot to seek care. The rural location and geographic spread of the community, augmented by lack of roads to access health facilities from remote kebeles may explain this association. Health centers are the nearest possible facilities which can provide TB microscopy and treatment, patients tend to seek care late unless they have a better means of transportation than to walk on foot or a place to stay close to where the health centers were. A similar association was found in a study conducted in southern Ethiopia, where a walking distance of >2 hours and places which require night travel contributed to delayed treatment seeking [21].

Excluding five hard to reach kebeles of the district from random selection of the study kebeles might have minimized the magnitude of patient delay, since access is among the predictors of delay. The other limitation of this study is the inability to obtain bacteriological tests to confirm whether symptomatic individuals were actually TB patients. This may affect comparability of the study with other studies that have actually recruited TB patients at health care facilities. In addition, sociocultural factors that can significantly affect treatment seeking behavior were not fully included. Therefore, further qualitative studies may provide a more complete picture of the determinant factors of patient delay in this community.

## Conclusion

Increasing awareness of the community about the risks of delayed care seeking and the importance of accessing health services close to the community can help decrease patient delay.

## Abbreviations

AOR: Adjusted Odds Ratio
COR: Crude Odds Ratio
CI: Confidence interval
DOTS: Directly Observed Therapy Short course,
HIV: Human Immuno Deficiency Virus
KNCV: Koninklijke Nederlandse Centrale Vereniging tot bestrijding der Tuberculose
LMIC: Low income and Middle Income Countries
TRAC: TB Research Advisory Committee
